# Post-choledochojejunostomy reflux cholangitis due to cholestasis in the blind end of the afferent limb dramatically improved by endoscopic ultrasound-guided gastroenteroscopy

**DOI:** 10.1055/a-2760-8857

**Published:** 2025-12-17

**Authors:** Kazunori Onuma, Susumu Hijioka, Yoshikuni Nagashio, Shota Harai, Daiki Yamashige, Joshua Josef Torres, Takuji Okusaka

**Affiliations:** 1 68380 Department of Hepatobiliary and Pancreatic Oncology, National Cancer Center Hospital, Tokyo, Japan; 2 Department of Internal Medicine, Silliman University Medical Center, Dumaguete City, Philippines

A 73-year-old woman had undergone pancreaticoduodenectomy with choledochojejunostomy 15 years earlier for intraductal papillary mucinous adenoma. Since the postoperative period, she had experienced recurrent episodes of cholangitis, occurring approximately twice per month and managed with antibiotics.

Computed tomography showed pneumobilia, endoscopy revealed a dilated anastomosis, and endoscopic retrograde cholangiopancreatography demonstrated no evidence of bile duct stenosis ( Fig. 1 **a–e** ). Hepatobiliary scintigraphy using 99mTc-N-pyridoxyl-5-methyltryptophan demonstrated tracer pooling in the blind end of the afferent limb without intrahepatic duct visualization ( Fig. 2 **a, b** ). Therefore, she was diagnosed with post-choledochojejunostomy reflux cholangitis (PCRC 1 2 ) due to cholestasis in the blind end. Endoscopic ultrasound-guided gastroenterostomy (EUS–GE) was planned to divert bile flow from the blind end to the stomach.

**Fig. 1:**
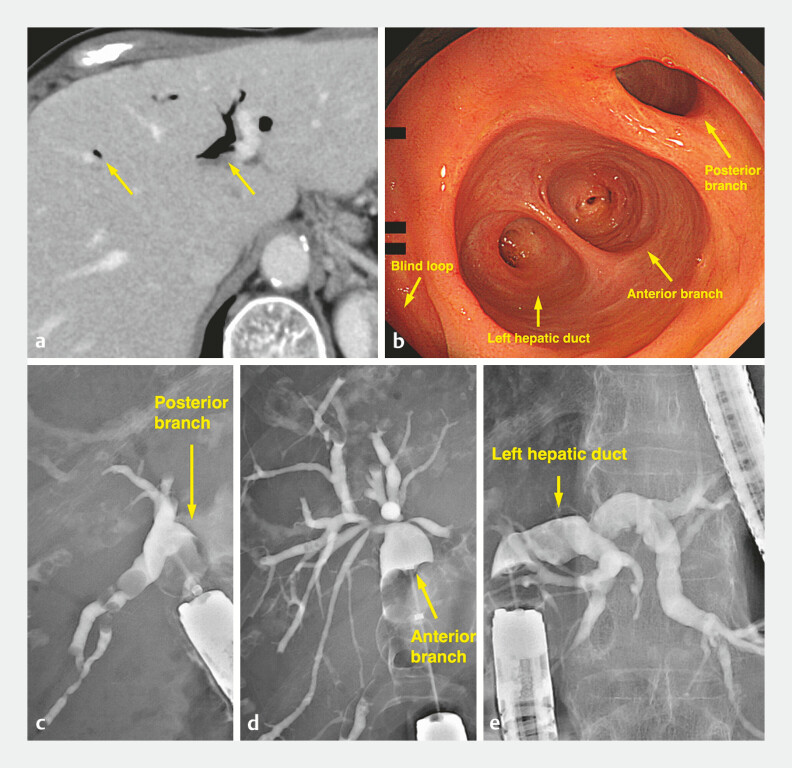
Imaging findings before the procedure. **a** Computed tomography (CT) showing pneumobilia (arrows) without bile duct dilatation. **b** Endoscopic image: The dilated choledochojejuno anastomosis. **c–e** Endoscopic retrograde cholangiography showing no stricture of the bile duct ( **c** right posterior branch, **d** right anterior branch, and **e** left hepatic duct).

**Fig. 2:**
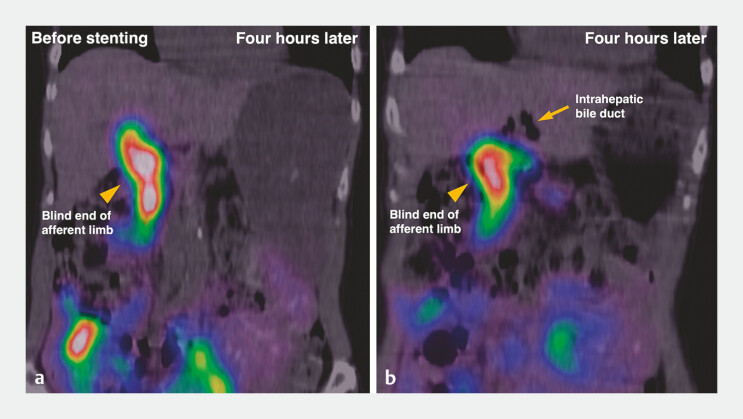
Hepatobiliary scintigraphy before stenting, on delayed imaging 4 hours later. **a, b** Tracer accumulation is seen in the blind end of the afferent limb (arrowhead), but not in the intrahepatic bile ducts (arrow).

A 7-Fr nasobiliary drainage tube was placed in the blind end, and saline was injected to visualize the target segment under EUS ( Fig. 3 **a, b** ). The blind end was punctured using a 19-gauge FNA needle, followed by guidewire insertion and contrast injection to confirm access into the intestinal lumen; then, the tract was dilated with a 4–mm balloon ( Fig. 4 **a** ). An 8 mm × 8 cm fully covered self-expandable metal stent was placed to bridge the blind end and the stomach ( Fig. 4 **b** ). A 7–Fr half-pigtail plastic stent was deployed inside for anchoring ( Fig. 4 **c, d** , Video 1 ). Follow-up scintigraphy showed marked tracer reduction in the blind end and flow into the stomach ( Fig. 5 **a–c** ). After the procedure, the frequency of cholangitis episodes decreased dramatically. She is currently under follow-up with regular stent exchanges every 6–12 months.

**Fig. 3:**
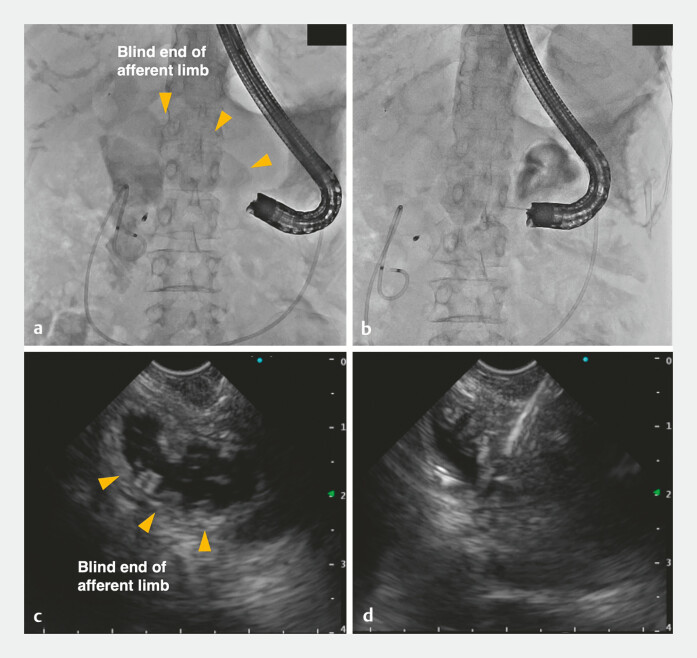
Procedure for puncturing the blind end of the afferent limb under endoscopic ultrasound (EUS) guidance. **a, b** Visualization of the target intestinal segment (arrowheads) under **a** EUS and **b** fluoroscopy by injecting saline through the nasobiliary drainage tube. **c, d** Puncture of the blind end of the afferent limb using a 19-gauge FNA needle.

**Fig. 4:**
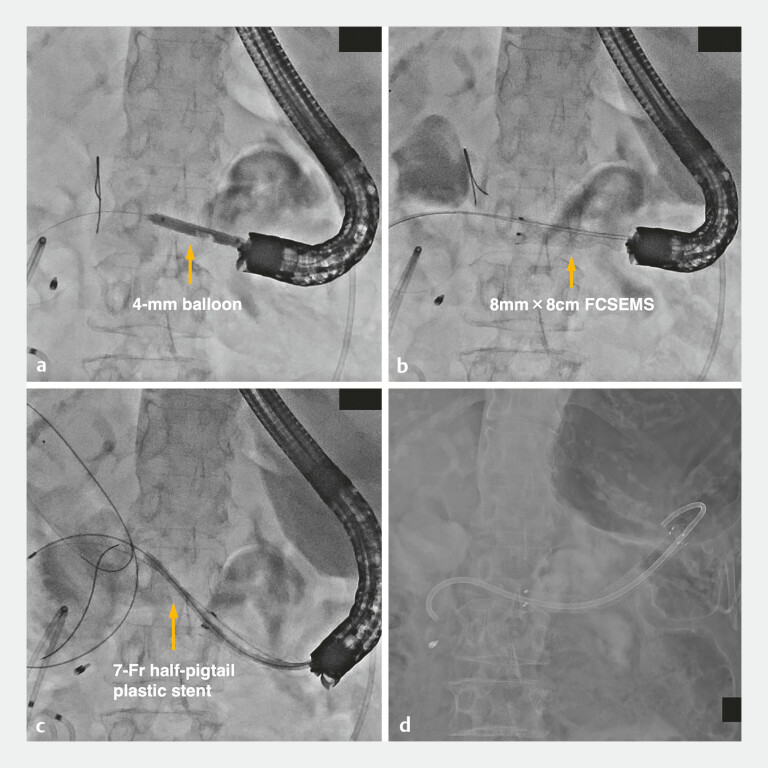
Procedure for placing a fully covered self-expandable metal stent (FCSEMS) and an anchor-type plastic biliary stent. **a** Dilation of the puncture site using a 4-mm balloon. **b–d** First, a FCSEMS was placed to bridge the blind end of the afferent limb and the stomach under fluoroscopic guidance ( **b** ). Second, a half-pigtail plastic stent was deployed inside the FCSEMS for anchoring ( **c, d** ).

EUS-GE in a patient with post-choledochojejunostomy reflux cholangitis due to cholestasis in the blind end of the afferent limb. EUS-GE, endoscopic ultrasound-guided gastroenterostomy.Video 1

**Fig. 5:**
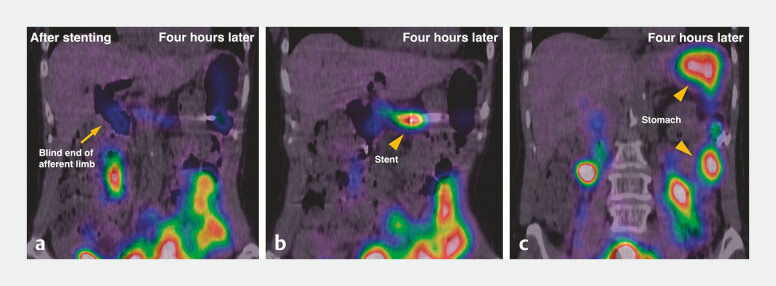
Hepatobiliary scintigraphy after stenting, on delayed imaging 4 hours later. **a** Marked tracer reduction in the blind end of the afferent limb (arrow). **b, c** Tracer flow from the blind end into the stomach through the stent (arrowheads).

Reflux cholangitis is a common complication of choledochojejunostomy 3 , but no definitive treatment has yet been established 4 . Although EUS-GE has shown effective and safe for afferent loop syndrome 5 , this is the first report demonstrating its efficacy for PCRC.

Endoscopy_UCTN_Code_TTT_1AS_2AI

## Notes

Endoscopy E-Videos https://eref.thieme.de/e-videos
*E-Videos* is an open access online section of the journal *Endoscopy* , reporting on interesting cases and new techniques in gastroenterological endoscopy. All papers include a high-quality video and are published with a Creative Commons CC-BY license. Endoscopy E-Videos qualify for HINARI discounts and waivers and eligibility is automatically checked during the submission process. We grant 100% waivers to articles whose corresponding authors are based in Group A countries and 50% waivers to those who are based in Group B countries as classified by Research4Life (see: https://www.research4life.org/access/eligibility/ ). This section has its own submission website at https://mc.manuscriptcentral.com/e-videos .

## References

[1] Koga T Hijioka S Ishikawa Y Duckbill-type antireflux self–expandable metal stent placement for post–choledochojejunostomy reflux cholangitis Endoscopy 2021 53 E174 E176 10.1055/a-1216-1220 32818991

[2] Maehara K Hijioka S Kawasaki Y A novel triple stenting in the treatment of post–choledochojejunostomy reflux cholangitis Endoscopy 2023 55 E191 E193 10.1055/a-1956-1055 36368667 PMC9829828

[3] Tocchi A Mazzoni G Liotta G Late development of bile duct cancer in patients who had biliary-enteric drainage for benign disease: a follow–up study of more than 1,000 patients Ann Surg 2001 234 210 214 11505067 10.1097/00000658-200108000-00011 PMC1422008

[4] Sanada Y Yamada N Taguchi M Recurrent cholangitis by biliary stasis due to non-obstructive afferent loop syndrome after pylorus-preserving pancreatoduodenectomy: report of a case Int Surg 2014 99 426 431 10.9738/INTSURG-D-13-00243.1 25058778 PMC4114374

[5] Hagiwara Y Hijioka S Nagashio Y Efficacy of endoscopic ultrasound-guided gastroenterostomy using self-expandable metallic stent for afferent loop syndrome: A single-center retrospective study J Gastroenterol Hepatol 2024 39 2136 2142 10.1111/jgh.16649 38845460

